# Cuterebral Ophthalmomyiasis Externa Presenting as Preseptal Cellulitis

**Published:** 2013-04-05

**Authors:** Yula A. Taormina, Caitlin Gannon, Josephine Nguyen, Jennifer Rhodes, Michael Foxworth, William Koch, Kelley Dodson

**Affiliations:** ^a^Department of Otolaryngology—Head and Neck Surgery; ^b^Division of Pediatric Infectious Diseases, Children's Hospital of Richmond; ^c^Division of Plastic and Reconstructive Surgery, Virginia Commonwealth University, Richmond

## DESCRIPTION

A 13-month-old Caucasian female presented with erythema, swelling, and pain of the left periorbital region. On admission, her vital signs were normal, and complete blood count did not reveal abnormalities. Blood and eye cultures were collected. On physical examination, the left eye was erythematous and edematous starting at the left medial canthus, tracking across the upper lid to the lateral angle of the eye, and extending inferiorly to involve the medial portion of the lower lid. A pinpoint sinus tract was visible on the medial aspect of the upper eyelid and was actively draining serosanguinous fluid. Head computed tomography demonstrated findings consistent with left preseptal orbital cellulitis and a small developing phlegmon. Patient was started on intravenous broad-spectrum antibiotics but failed to improve after 3 days. Incision and drainage were performed, which revealed a live 1-cm larva.

## QUESTIONS

1. **What is the diagnosis?**
2. **What are the organisms involved and the pathogenesis of this condition?**
3. **What is the most common clinical presentation of this condition? Which population is at highest risk of being affected?**
4. **What is the treatment?**

## DISCUSSION

Myiasis is the infestation of human or animal tissues by larval stage of flies.

Most cases of human myiasis in North America are caused by Dermatobia hominis, acquired in Central and South America by travelers. This insect, also known as the human botfly, has a peculiar life cycle, in which eggs are deposited on the body of a mosquito captured by the female fly. The eggs hatch in response to a change of temperature that occurs when the carrier insect attaches itself to a warm-blooded host. The hatched larva penetrates the skin, latching itself in the subcutaneous tissues for the next 30 to 40 days. It then emerges from the host tissues and burrows into soil to undergo the pupation stage, and metamorphose into an adult fly.^1^^-^4 Most cases of myiasis in North America are caused by fly larvae from South America (Dermatobia hominis) or Africa (Cordylobia anthropophaga), as these cases represent travelers returning from endemic regions. In North American patients without history of recent travel, this condition can present a diagnostic challenge. Cuterebra species are the most common causative agent of myiasis in patients residing in North America that do not have a travel history.^2^ Cuterebra species are large bee-resembling flies that are endemic in the Northeastern United States and Southeastern Canada. They are known obligate parasites of rodents, rabbits, and hares, and the eggs are normally laid in the leaves, stems, and underbrush located in the typical areas of habitat of these animals. First-stage larvae hatch from the eggs and invade the hosts via mucous membranes or by mouth, after which they migrate to the subcutaneous tissues for further development.^1^^,^^2^ According to Currier et al, Cuterebra possess specialized spinous processes that enable them to migrate through tissues.^3^ Direct-entry route from the surface of the skin is also possible. In fact, in 1958, Penner demonstrated this by experimentally infesting his own skin with Cuterebra larvae.^5^

Humans are considered to be accidental hosts, and infestation is an extremely rare event, which can occur when someone inadvertently comes in contact with eggs that are ready to hatch. Children are the most frequently affected group.^6^ The large majority (85%) of infections are cutaneous, and 15% involve viscera.^7^ Grossly, the usual human cutaneous lesion appears as an erythematous papule, 0.2- to 2-cm in diameter, with a central pore that may exude serous, serosanguinous, or purulent material. Occasionally, larvae may be seen intermittently protruding through the pore, as surface contact is required for respiration. The lesion is typically pruritic but may also be painful. It is often misdiagnosed as furuncle, cellulitis, or subcutaneous abscess. Of the visceral organs affected, ophthalmic infestation accounted for 70%, and 30% involved the upper respiratory tract.^7^ Infestation of the eyelid or periorbital tissues without globe invasion is termed external ophthalmomyiasis, a form of cutaneous disease.^3^ According to Delshad et al, 11 of 57 (19%) cases of cutaneous myiasis were found to involve the eyelid. This is to be distinguished from internal ophthalmomyiasis, which denotes the presence of larvae within the globe, either anterior or posterior portion.^3^ This is considered to be visceral disease.

Myiasis is a self-limited process, as larvae eventually spontaneously extrude from the skin to complete their life cycle outside the host. Theoretically, no treatment is necessary. However, expectant management is not favorable because of the physical discomfort, pain, and psychological distress experienced by the affected individual. Treatment is aimed at removing the larva from the skin, and several methods have been described in literature, all of which are identical to those used for extraction of the more commonly encountered Dermatobia hominis larvae. The least invasive method relies on the fact that the larvae are obligate aerobes and involves placing an obstructing substance over the central punctum. This will suffocate the larva, causing it to come out of its burrow in search of air, where it can be grasped and extracted with forceps. Paraffin, mineral oil, petroleum jelly, beeswax, glue and nail polish all have been used for this purpose, with success.^4^^,^^6^ Depending on the size and maturity of the larva, these noninvasive approaches may be difficult secondary to the anchoring spines. However, if successful, they are least likely to produce scarring.^8^ In cases of external ophthalmomyiasis, where periorbital tissues are affected, the location of the larval burrow is generally unfavorable to extrusion with forceps, and surgical excision is recommended. If the parasite invades into the globe or subretinal tissues (internal ophthalmomyiasis), laser treatment can be utilized to immobilize and destroy the organism with minimal risk of complications.^3^ Lidocaine injection into the base of the burrow can produce pressures high enough to push the larva out through the punctum onto the skin surface, and was employed by Nunzi and colleagues,^9^ allowing them to successfully expel 6 Dermatobia hominis larvae from their human hosts. In cases where standard noninvasive methods of extraction are ineffective and surgical intervention is contraindicated (superimposed bacterial cellulitis), commercial venom extractor can be used to rapidly and safely remove intact larvae from their burrows with negative pressure.^10^ Regardless of the extraction method used, great care must be taken not to damage the larva, thereby risking leaving small remains in the wound, as this will trigger a foreign body reaction with granuloma formation and severe secondary inflammation.

In summary, cuterebral external ophthalmomyiasis is a type of cutaneous larval disease. It can often present as an infectious process, and in our patient, appeared as preseptal cellulitis refractory to broad-spectrum antibacterial therapy. An interesting point about our case is that the child did not have a travel history and therefore falls into the category of having acquired ophthalmomyiasis in the United States (Williamsburg, Virginia). Cuterebra larva was the causative agent, which is consistent with the literature as being the most common cause of myiasis acquired in North America. Early clinical diagnosis is essential for prevention of unnecessary treatment, and it should be based primarily on patient's physical assessment and history of possible animal contact, rather than advanced imaging modalities. Most cases resolve without complications after removal of the offending organism and do not require antibiotic therapy.

## Figures and Tables

**Figure 1:**
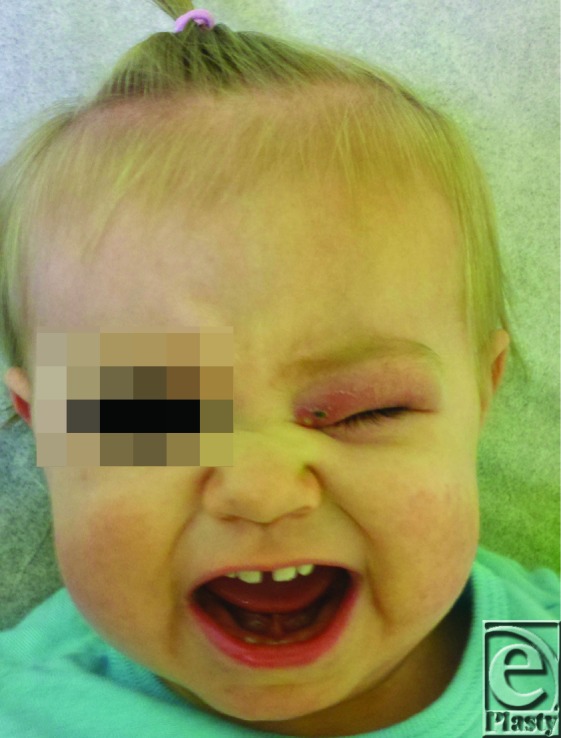
A 13-month-old child with periorbital inflammation caused by infestation with Cuterebra larva prior to surgical removal. The lesion was initially assumed to be preorbital cellulitis. Note the visible punctum in the center of a nodule at the medial canthus.

**Figure 2:**
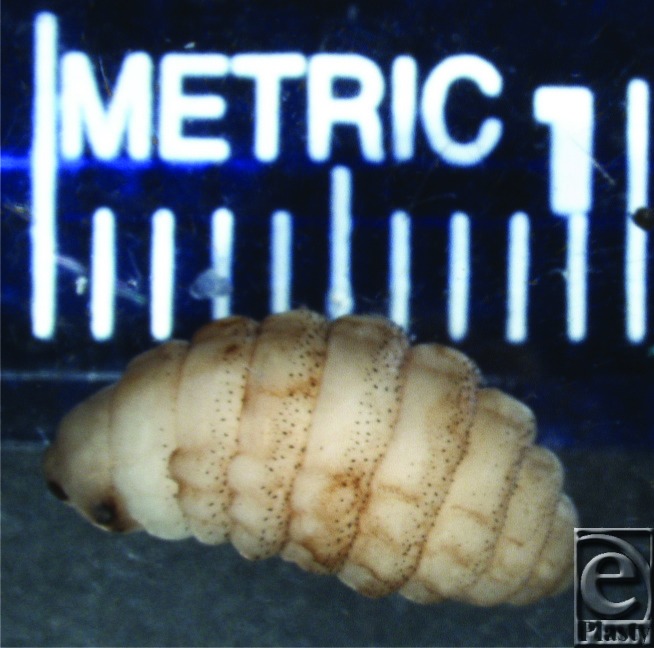
Second instar Cuterebra larva removed from the upper eyelid of the patient. Note that the body is covered with several concentric bands of spines arranged perpendicular to the longitudinal axis. Anchoring to host tissues is made possible with these appendages.

**Figure 3:**
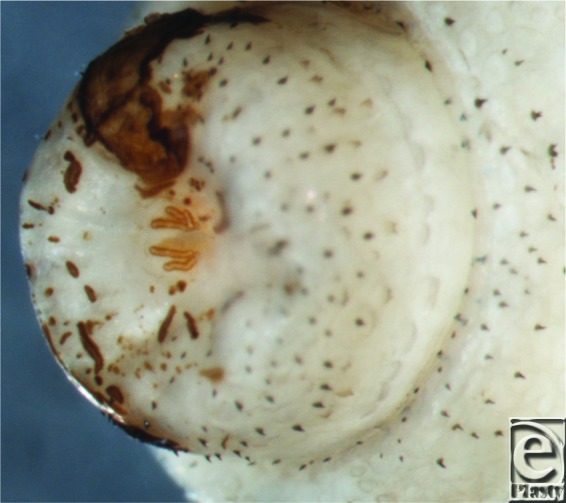
Magnification of posterior end of the larva demonstrating respiratory spiracles, essential for identification of Cuterebra species.

**Figure 4:**
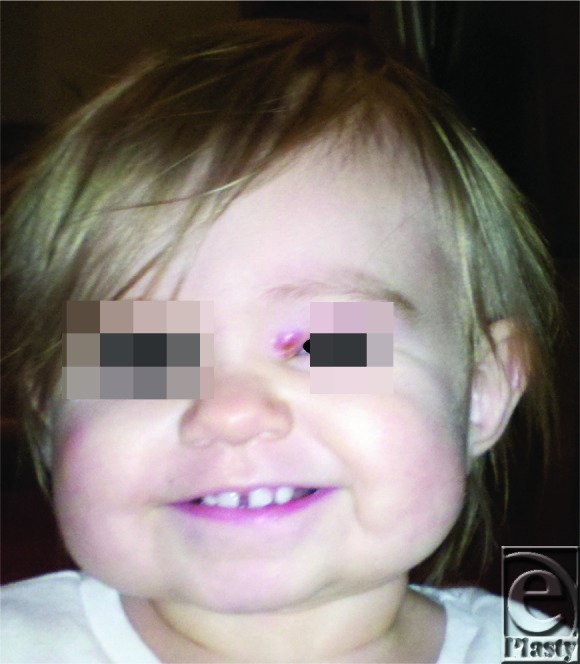
Photograph of the patient 2 days after surgical excision of larva demonstrates significant improvement, with only mild periorbital erythema and edema.
